# Effects of Household Processing on Rutin and Fagopyrin F in Common and Tartary Buckwheat

**DOI:** 10.3390/foods15183284

**Published:** 2026-09-17

**Authors:** Jimin Kim, Seong Ae Lee, Jaecheol Kim

**Affiliations:** Department of Food and Nutrition, Sungshin Women’s University, Seoul 01133, Republic of Korea

**Keywords:** common buckwheat, Tartary buckwheat, rutin, fagopyrin F, LC-MS/MS

## Abstract

Buckwheat contains rutin, a flavonoid, and fagopyrin F, a photosensitizer. This study compared household processing methods to determine how fagopyrin F content could be reduced while limiting rutin loss. Common and Tartary buckwheat (CB and TB) grains were processed using seven household methods: boiling, steaming, microwave heating, pan roasting, oven heating, deep frying, and air frying. Extractable phenolic and total flavonoid contents were determined colorimetrically; rutin and fagopyrin F were quantified by ultra-performance liquid chromatography with ultraviolet detection and tandem mass spectrometry, respectively. In CB, steaming and several dry-heating treatments increased the measured rutin content. In TB, microwave heating for 120 s, oven heating at 180 °C for 5 min, and air frying at 180 °C for 5 min maintained rutin at 95.7–105.9% of the control. All treatments reduced measured fagopyrin F content. The largest reductions occurred after medium-flame pan roasting, oven heating at 200 °C, deep frying at 190 °C, and air frying at 180–200 °C. Under corresponding processing conditions, the relative decrease in fagopyrin F was greater in CB than in TB. The rutin-to-fagopyrin F ratio increased under all calculable conditions. These results provide a basis for selecting processing conditions that reduce fagopyrin F with limited rutin loss.

## 1. Introduction

Buckwheat is a gluten-free pseudocereal in the family Polygonaceae and contains dietary fiber, proteins, and phenolic compounds [1,2]. Common buckwheat (CB; *Fagopyrum esculentum*) and Tartary buckwheat (TB; *Fagopyrum tataricum*) are the two main cultivated species. Archaeobotanical evidence indicates a cultivation history of several millennia in China. CB is widely grown in temperate regions of the Northern Hemisphere, whereas TB is principally cultivated in high-altitude regions, including the Himalayas [3]. Buckwheat grains are commonly dehulled for food use to produce groats, which can be milled into flour or used in other food products [1]. Rutin, a glycosylated flavonol, is one of the characteristic flavonoids in buckwheat. TB generally contains more rutin than CB [4,5]. Buckwheat grains are commonly consumed after processing; changes in the measured rutin content are therefore relevant to the compositional evaluation of buckwheat foods.

Buckwheat also contains fagopyrins, a group of naphthodianthrone photosensitizers structurally related to hypericin [6]. Fagopyrins occur mainly in flowers and other green tissues but have also been quantified in grains and grain-derived products [7,8]. Fagopyrin F is one of the major fagopyrins identified in both CB and TB tissues [9]. Fagopyrism has been reported primarily in animals following ingestion of fagopyrin-rich buckwheat materials and subsequent light exposure, whereas reports in humans are rare and quantitative human toxicity data remain limited [6]. Conversely, the photosensitizing activity of fagopyrin F has been investigated for antibacterial photodynamic applications. A fagopyrin F-rich fraction from Tartary buckwheat flowers generated reactive oxygen species under blue light and induced photodynamic inactivation of *Streptococcus mutans* and its biofilm [10]. However, given its potential phototoxicity, reducing residual fagopyrins during buckwheat processing may be desirable for food applications.

Food processing may alter measured phenolic contents through thermal degradation, enzymatic conversion, leaching, oxidation, matrix disruption, and changes in extractability [11,12]. Previous studies reported species- and method-dependent changes in phenolic profiles and rutin after boiling, steaming, microwave heating, and roasting of buckwheat [13,14]. Kočevar Glavač et al. [8] also reported lower total fagopyrin content after steaming TB grains. Direct comparisons of individual fagopyrin F and rutin under multiple household processing methods in both CB and TB remain limited.

Absolute contents of rutin and fagopyrin F describe the individual responses of these compounds to processing but do not indicate whether they change to the same extent. Stojilkovski et al. [7] compared the relative abundance of fagopyrins and flavonoids among buckwheat species and plant tissues. In this study, the rutin-to-fagopyrin F ratio was calculated to compare the relative changes in the two compounds during processing.

This study evaluated seven household processing methods in CB and TB grains. Extractable phenolic content (EPC) and total flavonoid content (TFC) were determined as complementary indices. Rutin and fagopyrin F were quantified by ultra-performance liquid chromatography with ultraviolet detection (UPLC-UV) and tandem mass spectrometry (UPLC-MS/MS), respectively. The study aimed to compare species- and treatment-dependent changes in rutin and fagopyrin F and to identify processing conditions that reduced fagopyrin F with limited rutin loss.

## 2. Materials and Methods

### 2.1. Chemicals and Reagents

Ethanol (99.5%), sodium carbonate, aluminum chloride, and potassium acetate were purchased from Samchun Pure Chemicals (Pyeongtaek, Republic of Korea). Folin–Ciocalteu reagent, gallic acid, quercetin, rutin, and LC-MS-grade formic acid were purchased from Sigma-Aldrich Co. (St. Louis, MO, USA). HPLC-grade acetonitrile was purchased from J.T. Baker (Phillipsburg, NJ, USA). 

### 2.2. Sample Preparation

Dehulled CB and TB grains were purchased from Bongpyeong Memil Farm (Pyeongchang, Republic of Korea). The moisture contents of untreated CB and TB grains, determined using the atmospheric-pressure oven-drying method, were 12.41 ± 0.12% and 5.98 ± 0.09%, respectively. Seven common household processing methods were applied to CB and TB grains. For each treatment, 20 g of grains were used, and the processing conditions are summarized in Table 1. Boiling was performed by immersing the grains in boiling water, whereas steaming was conducted using a domestic steamer. Microwave heating was performed using a microwave oven (MD-272CC, LG Electronics Co., Seoul, Republic of Korea) at 700 W. Pan roasting was conducted in a pan preheated for 1 min on a domestic gas stove at low or medium flame settings. Oven heating was performed using a conventional oven preheated for 5 min. Deep frying was conducted in soybean oil using a deep fryer, and air frying was performed using a preheated air fryer (HD9270, Philips Co., Seoul, Republic of Korea). 

### 2.3. Sample Extraction

Untreated and processed samples were freeze-dried, ground into powder, and stored at −20 °C. For the determination of EPC, TFC, rutin, and fagopyrin F, 1 g of each powdered sample was mixed with 10 mL of ethanol (99.5%) in a 50 mL conical tube with a screw cap (SPL, Seoul, Republic of Korea). The mixture was vortexed for 1 min and extracted in a water bath at 75 °C for 1 h without agitation. After extraction, the mixture was vortexed again for 1 min. The extracts were centrifuged (1236MG, GYROGEN Co., Gimpo, Republic of Korea) at 1004× *g* for 10 min and filtered through a 0.20 μm syringe filter. The filtrates were stored at −20 °C until analysis. EPC and TFC were determined in the ethanol extracts. The extraction did not include hydrolysis of the insoluble-bound fractions. All analytical results were expressed on a freeze-dried sample basis. 

### 2.4. Determination of Extractable Phenolic Content

EPC was determined according to Chen et al. [15] with modifications. An aliquot (20 μL) of each extract was mixed with 1.58 mL of distilled water and 100 μL of Folin–Ciocalteu reagent. After 5 min, 300 μL of 20% sodium carbonate was added, and the mixture was incubated in the dark for 30 min. Absorbance was measured at 765 nm using a microplate reader (SpectraMax Mini, Molecular Devices, San Jose, CA, USA). Gallic acid was used as the standard, and the results were expressed as mg of gallic acid equivalents (GAE)/g of freeze-dried sample.

### 2.5. Determination of Total Flavonoid Content

TFC was determined according to Chen et al. [15] with modifications. An aliquot (500 μL) of each extract was mixed with 1500 μL of 99.5% ethanol, 100 μL of 10% aluminum chloride, 100 μL of 1 M potassium acetate, and 2.8 mL of distilled water. After incubation at room temperature for 30 min, absorbance was measured at 415 nm using the microplate reader (Molecular Devices). Quercetin was used as the standard, and the results were expressed as mg of quercetin equivalents (QE)/g of freeze-dried sample.

### 2.6. Determination of Fagopyrin F and Rutin by UPLC-MS/MS and UPLC-UV

Fagopyrin F and rutin were separated under identical UPLC conditions based on previous studies [16,17]. Separation was performed using an ACQUITY UPLC H-Class system (Waters, Milford, MA, USA) equipped with an ACQUITY HSS T3 column (100 × 2.1 mm, 1.8 μm; Waters). The mobile phases were 0.1% formic acid in distilled water (A) and 0.1% formic acid in acetonitrile (B). Gradient elution was programmed as follows: 60% B at 0–0.21 min, 60–100% B at 0.21–1.46 min, 100% B at 1.46–2.92 min, 100–60% B at 2.92–3.54 min, and 60% B at 3.54–5.00 min. The flow rate was 0.3 mL/min, the injection volume was 5 μL, and the column temperature was 40 °C.

The previously validated UPLC-MS/MS method for fagopyrin F was applied under the same analytical conditions [17]. The fagopyrin F-rich fraction used as the reference material was prepared according to Kim et al. [17] and had a purity of 92.3 ± 0.1%. Fagopyrin F was quantified using a Xevo TQD triple-quadrupole mass spectrometer (Waters) operated in multiple reaction monitoring (MRM) mode. Electrospray ionization was performed in positive ion mode ([M+H]^+^), and the transition *m*/*z* 671.3 → 654.3 was used for quantification [17]. The capillary and cone voltages were 3.0 kV and 80 V, respectively. The source and desolvation temperatures were both 250 °C. The desolvation and cone gas flow rates were 1000 and 150 L/h, respectively, and the collision energy was 45 V. 

Rutin was separated under the same UPLC conditions and quantified using an ACQUITY TUV detector (Waters) at 353 nm. Chromatographic data were processed using MassLynx software version 4.1 (Waters).

### 2.7. Calculation of Relative Contents and Rutin-to-Fagopyrin F Ratios

The content of each compound in a processed sample relative to that in the corresponding untreated control was calculated as follows:

Relative content (%) = (C_processed_/C_control_) × 100 (1)

where C_processed_ and C_control_ represent the contents of the compound in the processed and untreated samples, respectively. The rutin-to-fagopyrin F ratio was calculated by dividing the mean rutin content by the mean fagopyrin F content on a mass basis. The ratio is dimensionless and was not multiplied by 100. When fagopyrin F was not detected, the ratio was recorded as not calculable (NC). Relative contents and ratios were descriptive values derived from the group means.

### 2.8. Statistical Analysis

Each processing treatment was independently performed in triplicate, and each replicate was extracted and analyzed separately. The results are expressed as mean ± standard deviation. Statistical analyses were performed separately for each buckwheat species using SPSS version 29.0.2.0 (IBM Corp., Armonk, NY, USA). Each processing condition was compared with the corresponding untreated control using an independent-samples *t*-test. Differences among conditions within each processing method were analyzed by one-way analysis of variance followed by Duncan’s multiple range test. Independent-samples *t*-tests were used for pan roasting and deep frying, each of which included two treatment conditions. Statistical significance was set at *p* < 0.05.

## 3. Results

### 3.1. Extractable Phenolic and Total Flavonoid Contents

The EPC and TFC values after household processing are presented in Table 2 and Table 3. The EPC values of untreated CB and TB were 1.87 and 5.64 mg GAE/g, respectively, and the corresponding TFC values were 2.00 and 6.55 mg QE/g. TB had numerically higher EPC and TFC values than CB under every condition, although the species were not compared statistically. In CB, boiling for 3–9 min reduced EPC to 1.24–1.33 mg GAE/g. Most treatments without direct water immersion increased the measured EPC. Deep frying produced the highest values, reaching 3.59 and 3.75 mg GAE/g at 160 and 190 °C, respectively. TFC remained near the control value under most conditions. Oven heating at 200 °C for 5 min and deep frying at both temperatures increased TFC, with deep-fried samples reaching approximately 4.10 mg QE/g. In TB, EPC decreased after boiling for 6 and 9 min, microwave heating for 150 s, and low-flame pan roasting. Boiling for 9 min resulted in the lowest EPC at 3.60 mg GAE/g. TFC decreased after boiling, both pan-roasting treatments, and selected steaming, microwave-heating, oven-heating, and air-frying conditions. Deep frying did not significantly affect EPC or TFC relative to the untreated control.

### 3.2. Rutin Contents

Rutin contents are presented in Table 4. Untreated CB and TB contained 65.35 and 5004.33 μg/g, respectively; the rutin content in TB was approximately 77-fold higher than that in CB. Boiling did not significantly affect the measured rutin content in CB, whereas steaming increased the measured content to 82.86–87.36 μg/g. Microwave heating for 30–90 s reduced rutin, whereas treatment for 120 and 180 s increased it to 74.05 and 83.14 μg/g, respectively. Medium-flame pan roasting increased rutin to 113.24 μg/g. Oven heating, deep frying, and air frying generally increased rutin with increasing treatment intensity. The highest value in CB was 116.79 μg/g after air frying at 200 °C for 10 min. In TB, boiling reduced rutin from 4083.27 μg/g after 3 min to 2543.80 μg/g after 9 min. Steaming also reduced rutin, and microwave heating caused significant reductions after 90–180 s. Medium-flame pan roasting and deep frying at 190 °C resulted in lower rutin contents than low-flame pan roasting and deep frying at 160 °C, respectively. The effects of oven heating and air frying depended on temperature. Rutin remained near or above the untreated value under several conditions at 140–180 °C but decreased at 200 °C, particularly after oven heating for 10 min.

### 3.3. Fagopyrin F Contents

Fagopyrin F contents are presented in Table 5. Untreated CB and TB contained 22.65 and 29.00 μg/g, respectively. Every processing condition reduced the measured fagopyrin F content relative to the corresponding untreated control. In CB, boiling reduced fagopyrin F from 11.03 μg/g after 3 min to 2.83 μg/g after 9 min. Steaming reduced the content to 3.21–3.95 μg/g. Microwave heating resulted in 2.15–5.88 μg/g. Fagopyrin F was not detected after medium-flame pan roasting, oven heating at 200 °C, or air frying at 180–200 °C. Deep frying reduced the content to approximately 2.1–2.3 μg/g. Fagopyrin F remained detectable in every processed TB sample. Its content generally decreased with increasing treatment time, temperature, or flame intensity. Microwave heating reduced it from 18.20 μg/g after 30 s to 7.68 μg/g after 180 s. Oven heating for 10 min reduced it from 19.40 μg/g at 140 °C to 2.58 μg/g at 200 °C. Medium-flame pan roasting and deep frying at 190 °C resulted in 4.94 and 3.52 μg/g, respectively.

### 3.4. Relative Contents and Rutin-to-Fagopyrin F Ratio

**Figure 1:**
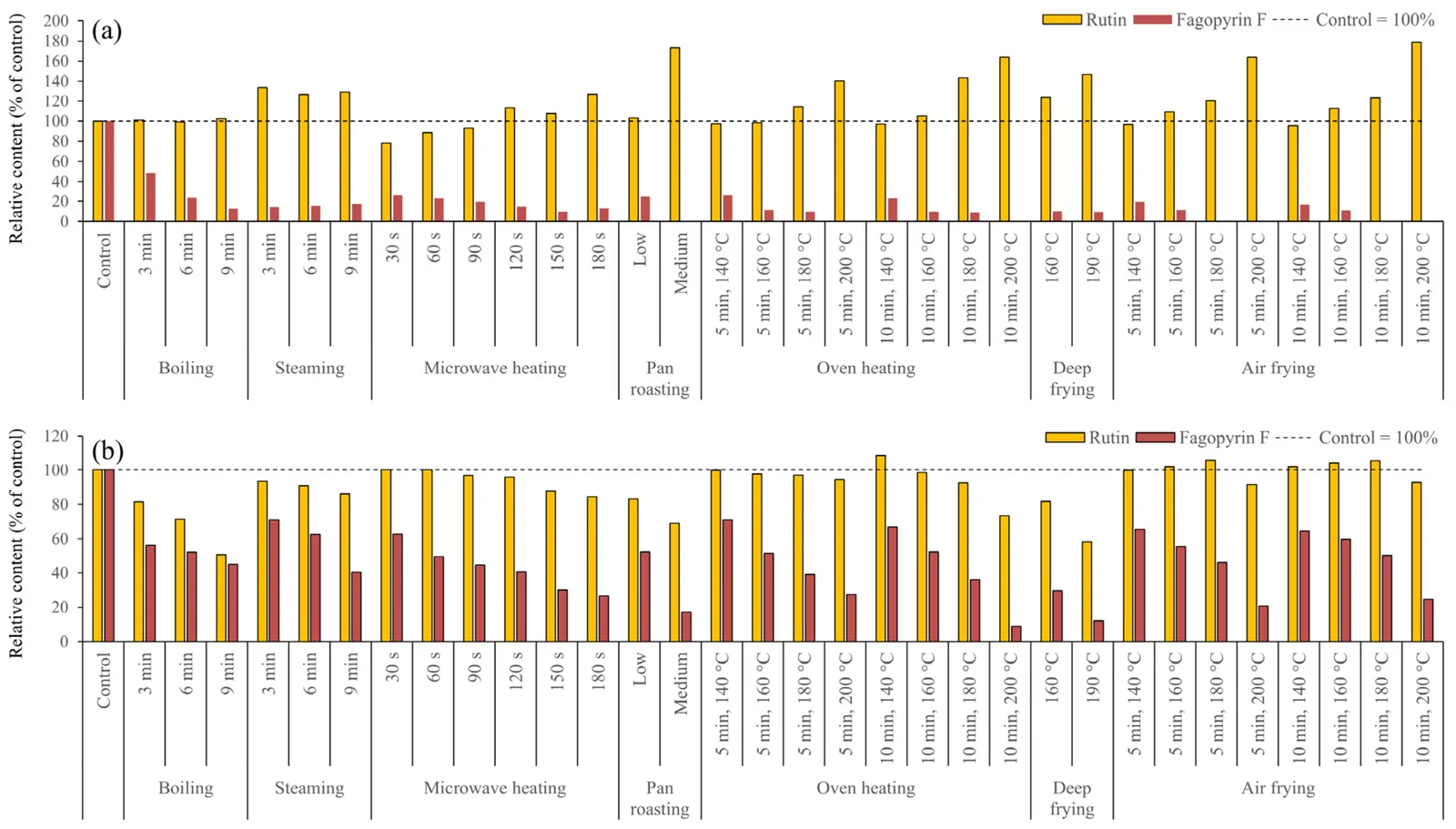
Relative rutin and fagopyrin F contents in common (**a**) and Tartary (**b**) buckwheat grains after household processing. Relative contents were calculated separately for each compound by dividing the processed group mean by the corresponding untreated group mean and multiplying by 100. The dashed line indicates the untreated control (100%). Bars are omitted where fagopyrin F was not detected. Values are descriptive calculations from group means.

**Figure 2:**
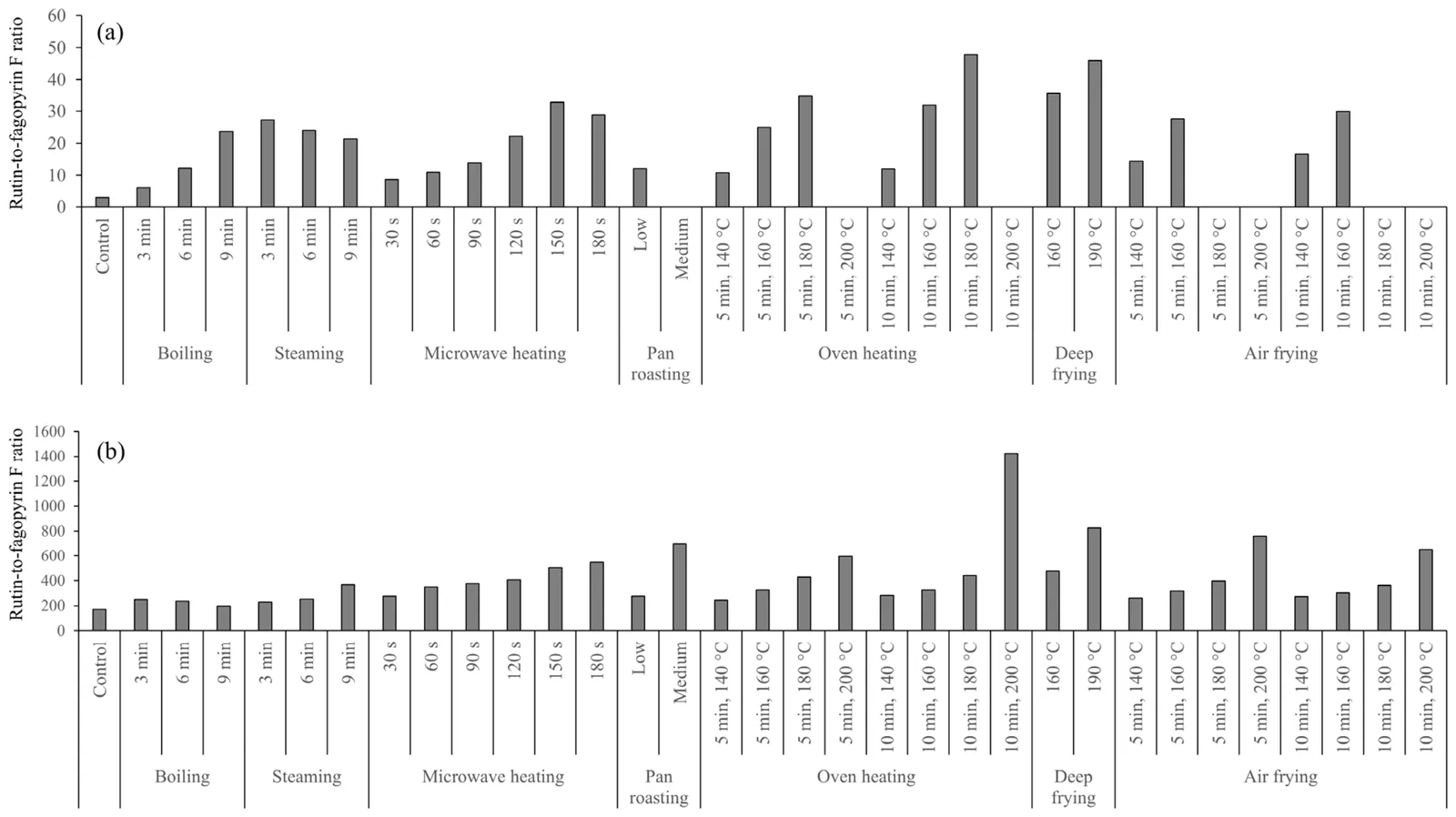
Rutin-to-fagopyrin F ratios in common (**a**) and Tartary (**b**) buckwheat grains after household processing. Ratios were calculated by dividing the mean rutin content by the mean fagopyrin F content on the same mass basis. Bars are omitted where the ratio could not be calculated because fagopyrin F was not detected. Values are descriptive calculations from group means. Note the different *y*-axis scales.

Relative rutin and fagopyrin F contents and their ratios are shown in Figure 1 and Figure 2, respectively. The calculated values are provided in Table S1. The ratios of untreated CB and TB were 2.89 and 172.6, respectively.

In CB, boiling maintained the measured rutin content at 99.2–102.5% of the control, whereas fagopyrin F decreased to 12.5–48.7%. The ratio consequently increased from 6.0 after 3 min to 23.7 after 9 min. After steaming, the rutin content was 126.8–133.7% of the control, whereas fagopyrin F was 14.2–17.4%, resulting in ratios of 21.4–27.2. Microwave heating resulted in rutin contents of 78.2–127.2% and fagopyrin F contents of 9.5–26.0% of their respective control values. Among treatments in which fagopyrin F remained quantifiable, the highest CB ratios were obtained after oven heating at 180 °C for 10 min (47.8) and deep frying at 190 °C (45.9). The ratio was not calculable after medium-flame pan roasting, oven heating at 200 °C, or air frying at 180–200 °C because fagopyrin F was not detected.

In TB, boiling reduced both compounds. Rutin decreased to 50.8–81.6% of the control, whereas fagopyrin F decreased to 44.9–56.2%. Steaming resulted in rutin contents of 86.0–93.5% and fagopyrin F contents of 40.4–70.7%. During microwave heating, rutin decreased from 100.1% of the control after 30 s to 84.3% after 180 s, whereas fagopyrin F decreased from 62.8% to 26.5%. The corresponding ratio increased from 275.2 to 549.1.

Five-minute oven heating maintained rutin at 94.5–99.9% of the TB control while reducing fagopyrin F to 27.4–71.1%. Air frying at 180–200 °C resulted in rutin contents of 91.4–105.9% and fagopyrin F contents of 20.7–50.3%. Oven heating at 200 °C for 10 min and deep frying at 190 °C produced the highest ratios, 1423.7 and 826.8, respectively, but rutin decreased to 73.4% and 58.2% of the control under these conditions.

## 4. Discussion

### 4.1. Processing-Dependent Changes in Extractable Phenolic and Total Flavonoid Contents

Boiling reduced EPC in CB and after 6 and 9 min in TB. Liu et al. [14] also reported phenolic losses in CB after thermal processing, including boiling. Transfer of soluble phenolics into the cooking water may contribute to the lower EPC in boiled grains. The absence of direct water immersion during pan roasting, oven heating, and air frying may account partly for their different EPC responses.

The EPC and TFC values represent assay-based measurements of the ethanol-extractable fraction. Martín-García et al. [18] analyzed solvent-extractable phenolics and phenolics released by alkaline hydrolysis separately in milling fractions obtained from dehulled buckwheat. Processing-induced changes in matrix structure may alter solvent accessibility and the measured EPC and TFC. Şensoy et al. [19] reported changes in extractable polar and nonpolar fractions after roasting CB flour at 200 °C, although the Folin–Ciocalteu phenolic content remained unchanged. Matrix disruption during heating may increase the recovery of assay-reactive compounds from processed CB.

Wronkowska et al. [20] reported the formation of Maillard reaction products during roasting of buckwheat groats. The Folin–Ciocalteu reagent also reacts with nonphenolic reducing compounds [21]. Heat-generated reducing compounds may therefore contribute to the measured EPC. The higher EPC in deep-fried CB may reflect changes in extractability and the amount of compounds that react with the reagent. 

EPC and TFC responded differently in TB. EPC remained comparatively stable under several dry-heating treatments, whereas TFC decreased after boiling, pan roasting, and selected microwave-heating, oven-heating, and air-frying conditions. Bhinder et al. [22] similarly reported that TFC in TB remained relatively stable after infrared roasting at 130 and 150 °C but decreased at 170 °C. Flavonoid stability during heating depends on molecular structure and the reaction environment. Buchner et al. [23] reported different degradation responses of rutin and quercetin during aqueous heating, with effects of pH and oxygen availability.

The approximately twofold increase in TFC in deep-fried CB exceeded the proportional increase in rutin content. The aluminum chloride response varies among flavonoid structures [24]. Changes in the composition and extractability of flavonoids may therefore contribute to the increased TFC response without a proportional increase in rutin.

Oil uptake was not quantified. Absorbed oil may contribute to the mass of the freeze-dried sample and lower the reported contents per gram, even without a change in the mass of the analyte. Changes after deep frying may therefore reflect both compound-specific responses and changes in sample composition.

### 4.2. Species-Dependent Changes in Rutin

The rutin response differed markedly between CB and TB. Steaming and several dry-heating treatments increased the measured rutin content in CB, whereas boiling, steaming, prolonged microwave heating, pan roasting, deep frying, and oven heating at 200 °C reduced rutin in TB.

The decrease in rutin during boiling and steaming in TB may involve aqueous transfer and enzymatic hydrolysis. TB contains endogenous rutinosidase, and hydration may increase contact between rutin and the enzyme before heat completely inactivates rutinosidase. Fujita and Yoshihashi [25] reported that heat treatment denatured rutinosidase in dehulled and gelatinized TB products. Kreft et al. [26] found lower rutin contents in common buckwheat noodles than in the dark flour used for their production and in precooked groats than in raw groats, and proposed rutin-degrading enzyme activity as a possible explanation. Kalinová et al. [27] detected rutin-degrading activity in intact TB achenes after heating at temperatures up to 140 °C. Rutin hydrolysis during wet processing may therefore occur after hydration and before complete rutinosidase inactivation.

The short microwave treatments (30 and 60 s) produced little change in TB rutin, whereas treatments for 90–180 s progressively reduced the content. Rapid initial heating may have limited the period of enzymatic hydrolysis. Longer microwave heating may have contributed to the lower measured rutin content. The simultaneous decrease in fagopyrin F with microwave time indicates that prolonged microwave treatment affected both compounds, although fagopyrin F decreased more extensively.

The effects of oven heating and air frying depended on processing intensity. Rutin remained near or above the untreated TB value under several conditions at 140–180 °C. Dry heating at 140–180 °C may inactivate rutinosidase and increase rutin extractability before direct thermal loss becomes dominant. Bhinder et al. [22] identified rutin as the most thermostable polyphenol among the compounds measured after infrared roasting of TB at 130–170 °C. In contrast, Noda et al. [28] reported progressive rutin losses with increasing roasting temperature and time. In this study, rutin remained near or above the control under several oven-heating and air-frying conditions at 140–180 °C but decreased after oven heating at 200 °C, medium-flame pan roasting, and deep frying at 190 °C.

The increased rutin content measured in processed CB may reflect greater accessibility to the extraction solvent. Roy et al. [29] reported changes in grain microstructure and starch organization after steam parboiling and hot soaking of CB grains, which were dehulled after treatment. Inglett et al. [30] found that phenolic and flavonoid recovery from commercial buckwheat flours depended on extraction conditions. Hydration during steaming and structural changes during heating may therefore increase the recovery of rutin that was less accessible in untreated grains. The same extraction procedure may yield different recoveries from untreated and processed matrices.

The opposite responses in CB and TB indicate that processing effects cannot be attributed to temperature alone. Initial rutin content, grain structure, hydration, endogenous enzyme activity, and heat-transfer rate may jointly influence the measured content after processing.

### 4.3. Reduction in Measured Fagopyrin F

Fagopyrin F decreased under every processing condition, whereas rutin remained unchanged or increased under several treatments. The two compounds therefore exhibited different responses to household processing.

Kočevar Glavač et al. [8] reported that steaming reduced total fagopyrins in TB grain, whereas bread making caused only a minor decrease. Kim et al. [31] reported that total fagopyrin contents, expressed as hypericin equivalents, were 92.5–95.7% lower in subcritical-water extracts of buckwheat leaves than in a methanol extract of untreated leaves. Although these studies did not quantify fagopyrin F individually, they demonstrate that buckwheat fagopyrins can decrease after steaming and during high-temperature aqueous processing.

In this study, fagopyrin F decreased during both wet and dry processing. Its marked reduction after steaming indicates that direct immersion in water was not required. During microwave and oven heating, the measured content generally decreased with increasing treatment time or temperature. In CB, fagopyrin F was not detected after medium-flame pan roasting, oven heating at 200 °C, or air frying at 180–200 °C. These patterns, together with the decrease reported during subcritical water treatment [31], are consistent with heat-induced loss or transformation of fagopyrins. The different responses reported after steaming and bread making [8] suggest that matrix structure, moisture distribution, and heat transfer may affect the extent of fagopyrin reduction.

Untreated CB and TB contained 22.65 and 29.00 μg/g of fagopyrin F, respectively. Fagopyrin F decreased proportionally more in CB than in TB under conditions where it remained detectable in both species. Under the remaining conditions, fagopyrin F was not detected in CB and remained detectable in TB. Common and Tartary buckwheat differ in the protein, starch, lipid, and dietary-fiber composition of their flour and bran [32]. These differences may affect heat transfer, matrix disruption, and the association or extractability of fagopyrin F within the grain. The greater decrease in CB may therefore reflect species-specific matrix effects rather than the initial fagopyrin F content alone.

Fagopyrin F also decreased under conditions that caused limited changes in rutin. In TB, rutin remained near the control level after microwave heating for 120 s, oven heating at 180 °C for 5 min, and air frying at 180 °C for 5 min, whereas fagopyrin F decreased markedly. In CB, steaming increased the measured rutin content while reducing fagopyrin F. These responses are consistent with lower processing stability of fagopyrin F than rutin under the tested conditions.

Previous studies evaluated total fagopyrins after steaming or high-temperature extraction but did not compare individual fagopyrin F across multiple household processing methods in both CB and TB. To our knowledge, this is the first study to compare changes in fagopyrin F under seven household processing methods in both species.

The MRM analysis quantified parent fagopyrin F and did not characterize processing-derived products with different precursor ions. Son et al. [33] used full-scan Orbitrap mass spectrometry and MS/MS fragmentation to characterize structurally related soyasaponins. Liquid chromatography–high-resolution tandem mass spectrometry (LC–HRMS/MS) may similarly help investigate processing-derived fagopyrin products.

### 4.4. Interpretation of Relative Contents and the Rutin-to-Fagopyrin F Ratio

The relative content data clarify how the rutin-to-fagopyrin F ratio changed during processing. A higher ratio can result from a marked decrease in fagopyrin F with little change in rutin or from concurrent decreases in both compounds when fagopyrin F decreases to a greater extent. Accordingly, the ratio alone does not distinguish these two responses, and the relative contents of both compounds are also required.

In CB, the increases in the ratio were mainly associated with substantial reductions in fagopyrin F while the measured rutin content remained stable or increased. In TB, the largest ratios occurred under conditions that also reduced rutin. Thus, the highest ratio did not necessarily correspond to the greatest rutin preservation.

The ratio can be used to compare processing selectivity within each species and to identify treatments that reduce fagopyrin F with limited rutin loss. These treatments can be evaluated further by considering processing yield, sensory properties, and the contents of other fagopyrins and related compounds. Because untreated CB and TB differed markedly in their initial rutin contents, the ratio is more appropriate for comparing treatments within each species than for ranking the two species directly.

### 4.5. Visual Changes During Dry Heating

**Figure 3:**
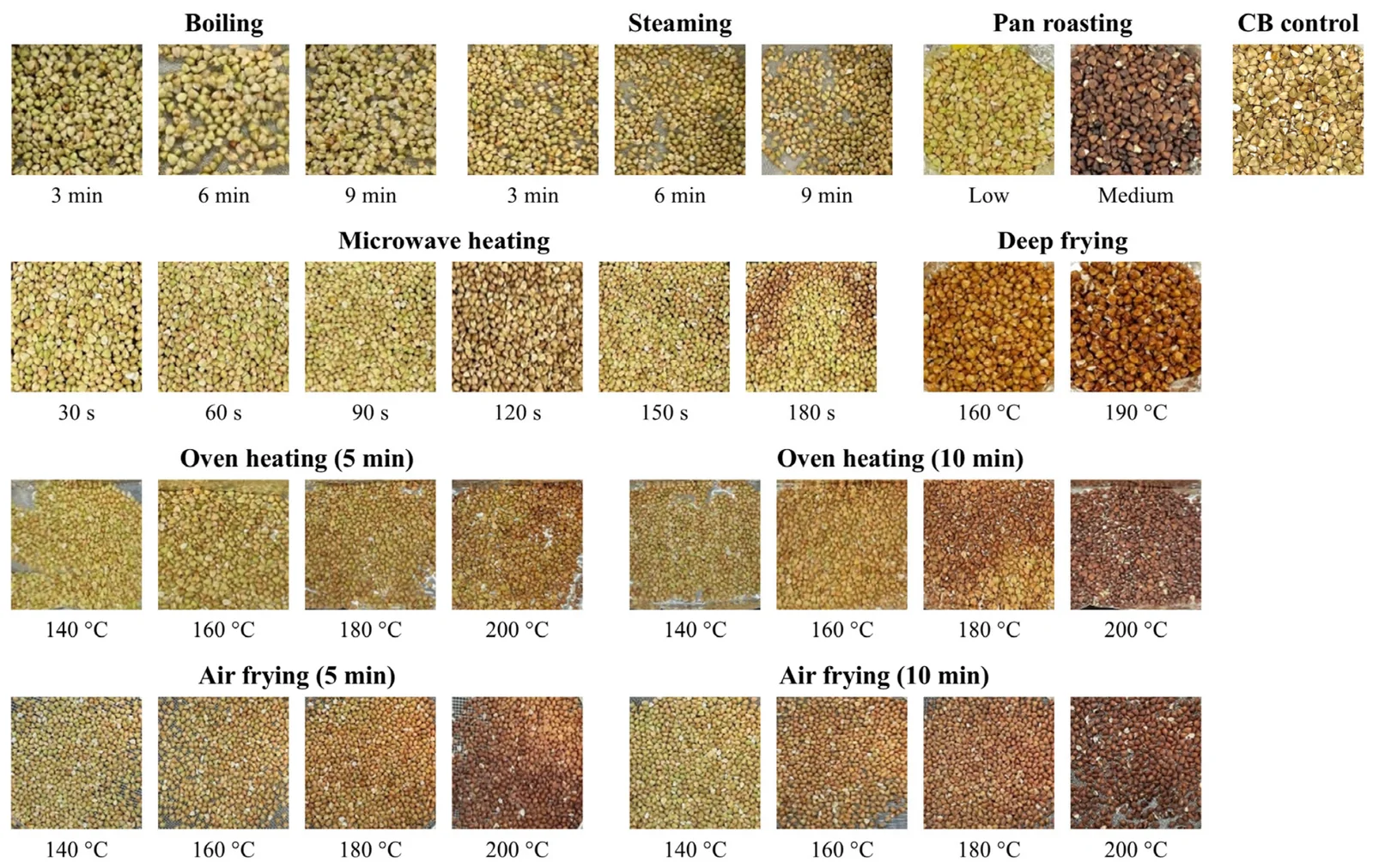
Visual appearance of common buckwheat grains after household processing. Processing conditions are indicated in the figure.

**Figure 4:**
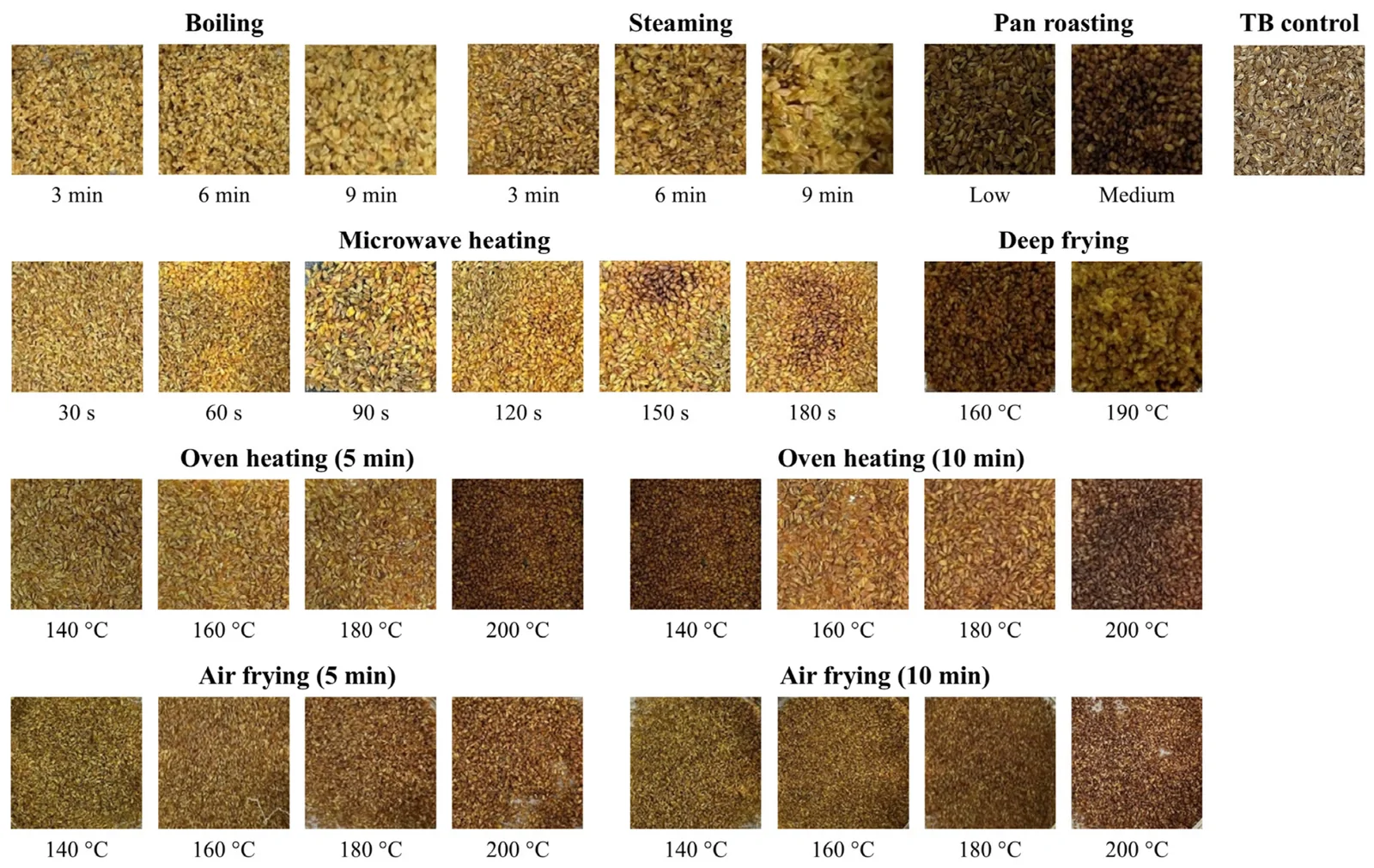
Visual appearance of Tartary buckwheat grains after household processing. Processing conditions are indicated in the figure.

The grains appeared darker at higher oven-heating and air-frying temperatures and after medium-flame pan roasting and deep frying (Figure 3 and Figure 4). Similar decreases in lightness and increases in thermal browning have been reported during buckwheat roasting [22,28].

## 5. Conclusions

Household processing reduced the measured fagopyrin F content under every tested condition, whereas rutin responses differed between CB and TB. In CB, steaming and several dry-heating treatments maintained or increased the measured rutin content. In TB, microwave heating for 120 s, oven heating at 180 °C for 5 min, and air frying at 180 °C for 5 min maintained rutin near the control level while reducing fagopyrin F, whereas oven heating at 200 °C for 10 min and deep frying at 190 °C caused greater rutin loss. The rutin-to-fagopyrin F ratio increased under all calculable conditions. The highest ratios did not correspond to the highest rutin contents. These results provide a basis for selecting species-specific processing conditions that reduce fagopyrin F with limited rutin loss.

## Figures and Tables

**Table 1:** Household processing methods and treatment conditions applied to common and Tartary buckwheat grains.

Processing Method	Equipment or Heating Medium	Processing Condition	Time
Boiling	Boiling water	100 °C	3, 6, 9 min
Steaming	Domestic steamer	Steam	3, 6, 9 min
Microwave heating	Microwave oven	700 W	30, 60, 90, 120, 150, 180 s
Pan roasting	Preheated pan on a domestic gas stove	Low or medium flame settings	2 min
Oven heating	Conventional oven	140, 160, 180, 200 °C	5, 10 min
Deep frying	Soybean oil	160, 190 °C	3 min
Air frying	Air fryer	140, 160, 180, 200 °C	5, 10 min

Untreated grains were used as controls. Each processing treatment was performed using 20 g of CB or TB grains.

**Table 2:** Extractable phenolic content of common and Tartary buckwheat grains after household processing.

Processing Methods and Treatment Conditions			Extractable Phenolic Content (mg GAE/g)	
			Common Buckwheat	Tartary Buckwheat
Control	–		1.87 ± 0.12	5.64 ± 0.41
Boiling	3 min		1.32 ± 0.05 ^a^**	5.18 ± 0.71 ^b^
	6 min		1.33 ± 0.13 ^a^**	4.64 ± 0.35 ^b^*
	9 min		1.24 ± 0.03 ^a^***	3.60 ± 0.06 ^a^**
Steaming	3 min		2.32 ± 0.16 ^b^*	5.36 ± 0.31 ^a^
	6 min		2.33 ± 0.06 ^b^**	5.52 ± 0.17 ^a^
	9 min		2.02 ± 0.06 ^a^	4.92 ± 0.45 ^a^
Microwave heating	30 s		2.32 ± 0.14 ^a^*	5.97 ± 0.35 ^c^
	60 s		2.29 ± 0.02 ^a^**	5.73 ± 0.31 ^bc^
	90 s		2.54 ± 0.10 ^ab^**	5.33 ± 0.09 ^abc^
	120 s		2.38 ± 0.09 ^a^**	5.39 ± 0.49 ^abc^
	150 s		2.74 ± 0.20 ^b^**	4.77 ± 0.24 ^a^*
	180 s		2.48 ± 0.27 ^ab^*	5.22 ± 0.44 ^ab^
Pan roasting	Low flame		2.56 ± 0.16 **	4.81 ± 0.14 *
	Medium flame		2.64 ± 0.10 ***	5.15 ± 0.33
Oven heating	5 min	140 °C	2.34 ± 0.10 ^a^**	5.32 ± 0.35 ^a^
		160 °C	2.50 ± 0.19 ^a^**	5.23 ± 0.62 ^a^
		180 °C	2.61 ± 0.22 ^a^**	5.24 ± 0.17 ^a^
		200 °C	3.26 ± 0.36 ^b^**	5.47 ± 0.22 ^a^
	10 min	140 °C	2.41 ± 0.10 ^b^**	6.00 ± 0.48 ^a^
		160 °C	2.17 ± 0.09 ^a^*	5.63 ± 0.43 ^a^
		180 °C	2.60 ± 0.10 ^b^**	5.48 ± 0.24 ^a^
		200 °C	2.39 ± 0.14 ^b^**	5.50 ± 0.20 ^a^
Deep frying		160 °C	3.59 ± 0.33 ***	6.27 ± 0.61
		190 °C	3.75 ± 0.05 ***	5.28 ± 0.12
Air frying	5 min	140 °C	2.44 ± 0.23 ^a^*	6.02 ± 0.36 ^b^
		160 °C	2.38 ± 0.11 ^a^**	5.62 ± 0.43 ^ab^
		180 °C	2.42 ± 0.09 ^a^**	5.96 ± 0.27 ^b^
		200 °C	2.94 ± 0.19 ^b^**	5.13 ± 0.43 ^a^
	10 min	140 °C	2.50 ± 0.27 ^a^*	5.53 ± 0.35 ^a^
		160 °C	2.57 ± 0.07 ^a^***	6.07 ± 0.52 ^a^
		180 °C	2.78 ± 0.26 ^a^**	5.97 ± 0.36 ^a^
		200 °C	2.43 ± 0.20 ^a^*	5.92 ± 0.36 ^a^

Values are means ± standard deviations (*n* = 3). EPC was determined in the ethanol-extractable fraction and expressed as mg GAE per gram of freeze-dried sample. Different lowercase superscript letters within each processing method and species indicate significant differences among treatment conditions (*p* < 0.05, one-way ANOVA followed by Duncan’s multiple range test). For oven heating and air frying, comparisons were conducted among temperatures at each processing time. Asterisks indicate significant differences from the corresponding untreated control (* *p* < 0.05, ** *p* < 0.01, and *** *p* < 0.001; independent-samples *t*-test). CB, common buckwheat; TB, Tartary buckwheat; GAE, gallic acid equivalents.

**Table 3:** Total flavonoid content of common and Tartary buckwheat grains after household processing.

Processing Methods and Treatment Conditions			Total Flavonoid Content (mg QE/g)	
			Common Buckwheat	Tartary Buckwheat
Control	–		2.00 ± 0.09	6.55 ± 0.48
Boiling	3 min		2.02 ± 0.17 ^a^	5.10 ± 0.24 ^b^**
	6 min		2.04 ± 0.10 ^a^	4.57 ± 0.16 ^ab^**
	9 min		2.19 ± 0.16 ^a^	3.93 ± 0.06 ^a^**
Steaming	3 min		2.00 ± 0.12 ^a^	5.52 ± 0.21 ^a^*
	6 min		2.07 ± 0.04 ^a^	5.76 ± 0.39 ^a^
	9 min		2.12 ± 0.12 ^a^	5.26 ± 0.70 ^a^
Microwave heating	30 s		1.96 ± 0.14 ^a^	5.68 ± 0.40 ^a^
	60 s		2.05 ± 0.11 ^a^	5.70 ± 0.36 ^a^
	90 s		2.01 ± 0.13 ^a^	5.56 ± 0.19 ^a^*
	120 s		2.10 ± 0.07 ^a^	5.12 ± 0.88 ^a^
	150 s		2.08 ± 0.14 ^a^	4.82 ± 0.42 ^a^**
	180 s		2.02 ± 0.06 ^a^	5.01 ± 0.45 ^a^*
Pan roasting	Low flame		2.10 ± 0.22	4.81 ± 0.31 **
	Medium flame		2.02 ± 0.14	4.76 ± 0.30 **
Oven heating	5 min	140 °C	2.11 ± 0.03 ^a^	5.55 ± 0.53 ^a^
		160 °C	2.01 ± 0.20 ^a^	5.41 ± 0.37 ^a^*
		180 °C	1.98 ± 0.18 ^a^	5.66 ± 0.42 ^a^
		200 °C	2.24 ± 0.10 ^a^*	5.82 ± 0.10 ^a^
	10 min	140 °C	2.00 ± 0.04 ^ab^	6.39 ± 0.90 ^a^
		160 °C	1.93 ± 0.10 ^a^	5.74 ± 0.46 ^a^
		180 °C	2.13 ± 0.11 ^b^	5.33 ± 0.55 ^a^*
		200 °C	2.01 ± 0.10 ^ab^	5.54 ± 0.47 ^a^
Deep frying		160 °C	4.11 ± 0.15 ***	6.77 ± 0.83
		190 °C	4.10 ± 0.27 ***	6.12 ± 0.17
Air frying	5 min	140 °C	2.01 ± 0.19 ^a^	6.70 ± 0.48 ^b^
		160 °C	2.04 ± 0.15 ^a^	5.56 ± 0.38 ^a^*
		180 °C	1.93 ± 0.14 ^a^	6.41 ± 0.57 ^b^
		200 °C	1.88 ± 0.14 ^a^	4.81 ± 0.24 ^a^**
	10 min	140 °C	2.01 ± 0.11 ^a^	5.53 ± 0.40 ^a^*
		160 °C	2.09 ± 0.14 ^a^	6.06 ± 0.23 ^a^
		180 °C	2.07 ± 0.17 ^a^	6.29 ± 0.39 ^a^
		200 °C	1.86 ± 0.10 ^a^	5.63 ± 0.50 ^a^

Values are means ± standard deviations (*n* = 3). TFC was determined in the ethanol-extractable fraction and expressed as mg QE per gram of freeze-dried sample. Different lowercase superscript letters within each processing method and species indicate significant differences among treatment conditions (*p* < 0.05, one-way ANOVA followed by Duncan’s multiple range test). For oven heating and air frying, comparisons were conducted among temperatures at each processing time. Asterisks indicate significant differences from the corresponding untreated control (* *p* < 0.05, ** *p* < 0.01, and *** *p* < 0.001; independent-samples *t*-test). CB, common buckwheat; TB, Tartary buckwheat; QE, quercetin equivalents.

**Table 4:** Rutin contents in common and Tartary buckwheat grains after household processing.

Processing Methods and Treatment Conditions			Rutin (µg/g)	
			Common Buckwheat	Tartary Buckwheat
Control	–		65.35 ± 0.71	5004.33 ± 53.17
Boiling	3 min		66.10 ± 0.49 ^a^	4083.27 ± 117.90 ^c^***
	6 min		64.83 ± 2.38 ^a^	3575.70 ± 151.90 ^b^***
	9 min		66.96 ± 2.86 ^a^	2543.80 ± 100.95 ^a^***
Steaming	3 min		87.36 ± 0.29 ^b^***	4679.12 ± 13.47 ^c^***
	6 min		82.86 ± 2.49 ^a^***	4542.81 ± 89.41 ^b^**
	9 min		84.60 ± 1.87 ^ab^***	4305.80 ± 65.68 ^a^***
Microwave heating	30 s		51.13 ± 3.12 ^a^**	5009.09 ± 88.21 ^d^
	60 s		57.88 ± 1.12 ^b^***	5016.36 ± 85.04 ^d^
	90 s		60.69 ± 0.68 ^b^**	4851.38 ± 33.69 ^c^*
	120 s		74.05 ± 1.76 ^c^**	4788.15 ± 32.86 ^c^**
	150 s		70.47 ± 3.81 ^c^	4399.15 ± 54.69 ^b^***
	180 s		83.14 ± 3.93 ^d^**	4217.36 ± 106.00 ^a^***
Pan roasting	Low flame		67.40 ± 2.52	4170.03 ± 101.41 ***
	Medium flame		113.24 ± 1.58 ***^,###^	3457.99 ± 69.13 ***^,###^
Oven heating	5 min	140 °C	63.57 ± 1.47 ^a^	4997.45 ± 58.24 ^b^
		160 °C	64.31 ± 1.45 ^a^	4894.16 ± 87.47 ^b^
		180 °C	74.65 ± 2.35 ^b^**	4860.71 ± 81.84 ^ab^
		200 °C	91.86 ± 2.47 ^c^***	4727.82 ± 86.14 ^a^**
	10 min	140 °C	63.30 ± 1.46 ^a^	5440.30 ± 132.72 ^d^**
		160 °C	68.62 ± 2.30 ^b^	4931.98 ± 69.12 ^c^
		180 °C	93.73 ± 1.67 ^c^***	4635.50 ± 63.29 ^b^**
		200 °C	107.16 ± 1.38 ^d^***	3673.04 ± 67.01 ^a^***
Deep frying		160 °C	80.81 ± 1.24 ***	4087.04 ± 41.42 ***
		190 °C	95.96 ± 1.37 ***^,###^	2910.28 ± 53.33 ***^,###^
Air frying	5 min	140 °C	63.29 ± 1.39 ^a^	5000.93 ± 30.21 ^b^
		160 °C	71.30 ± 1.27 ^b^**	5095.34 ± 82.26 ^bc^
		180 °C	78.53 ± 0.66 ^c^***	5297.83 ± 175.79 ^c^
		200 °C	107.20 ± 1.42 ^d^***	4572.77 ± 101.52 ^a^**
	10 min	140 °C	62.39 ± 1.71 ^a^	5097.60 ± 81.30 ^b^
		160 °C	73.45 ± 1.23 ^b^***	5228.69 ± 85.07 ^bc^*
		180 °C	80.40 ± 1.17 ^c^***	5288.46 ± 96.40 ^c^*
		200 °C	116.79 ± 0.95 ^d^***	4640.39 ± 112.59 ^a^**

Values are means ± standard deviations (*n* = 3). All values are expressed on a freeze-dried basis. Different lowercase superscript letters within each processing method and species indicate significant differences among treatment conditions (*p* < 0.05, one-way ANOVA followed by Duncan’s multiple range test). For oven heating and air frying, comparisons were conducted among temperatures at each processing time. Asterisks indicate significant differences from the corresponding untreated control (* *p* < 0.05, ** *p* < 0.01, and *** *p* < 0.001; independent-samples *t*-test). Hash symbols indicate significant differences between the two pan-roasting or deep-frying conditions (^###^ *p* < 0.001; independent-samples *t*-test). CB, common buckwheat; TB, Tartary buckwheat.

**Table 5:** Fagopyrin F contents in common and Tartary buckwheat grains after household processing.

Processing Methods and Treatment Conditions			Fagopyrin F (μg/g)	
			Common Buckwheat	Tartary Buckwheat
Control	–		22.65 ± 0.65	29.00 ± 1.06
Boiling	3 min		11.03 ± 1.78 ^c^***	16.30 ± 0.71 ^b^***
	6 min		5.32 ± 0.75 ^b^***	15.10 ± 0.67 ^b^***
	9 min		2.83 ± 0.85 ^a^***	13.02 ± 0.57 ^a^***
Steaming	3 min		3.21 ± 0.67 ^a^***	20.50 ± 0.85 ^c^***
	6 min		3.45 ± 0.75 ^a^***	18.10 ± 1.04 ^b^***
	9 min		3.95 ± 0.19 ^a^***	11.70 ± 0.28 ^a^***
Microwave heating	30 s		5.88 ± 0.28 ^d^***	18.20 ± 0.75 ^f^***
	60 s		5.26 ± 0.49 ^cd^***	14.40 ± 0.19 ^e^***
	90 s		4.39 ± 0.60 ^c^***	12.90 ± 0.56 ^d^***
	120 s		3.33 ± 0.39 ^b^***	11.80 ± 0.37 ^c^***
	150 s		2.15 ± 0.28 ^a^***	8.73 ± 0.47 ^b^***
	180 s		2.89 ± 0.75 ^ab^***	7.68 ± 0.56 ^a^***
Pan roasting	Low flame		5.57 ± 0.75 ***	15.20 ± 1.03 ***
	Medium flame		ND	4.94 ± 0.39 ***^,###^
Oven heating	5 min	140 °C	5.88 ± 0.47 ^c^***	20.60 ± 0.47 ^d^***
		160 °C	2.58 ± 0.43 ^b^***	15.00 ± 0.67 ^c^***
		180 °C	2.15 ± 0.22 ^b^***	11.30 ± 0.84 ^b^***
		200 °C	ND	7.93 ± 0.60 ^a^***
	10 min	140 °C	5.26 ± 0.37 ^c^***	19.40 ± 0.60 ^d^***
		160 °C	2.15 ± 0.39 ^b^***	15.20 ± 0.57 ^c^***
		180 °C	1.96 ± 0.11 ^b^***	10.50 ± 0.49 ^b^***
		200 °C	ND	2.58 ± 0.28 ^a^***
Deep frying		160 °C	2.27 ± 0.19 ***	8.55 ± 0.39 ***
		190 °C	2.09 ± 0.19 ***	3.52 ± 0.28 ***^,###^
Air frying	5 min	140 °C	4.39 ± 0.47 ^c^***	19.10 ± 0.49 ^d^***
		160 °C	2.58 ± 0.22 ^b^***	16.10 ± 0.37 ^c^***
		180 °C	ND	13.30 ± 0.78 ^b^***
		200 °C	ND	6.00 ± 0.49 ^a^***
	10 min	140 °C	3.76 ± 0.49 ^c^***	18.70 ± 0.28 ^d^***
		160 °C	2.46 ± 0.37 ^b^***	17.30 ± 0.28 ^c^***
		180 °C	ND	14.60 ± 0.19 ^b^***
		200 °C	ND	7.12 ± 0.49 ^a^***

Values are means ± standard deviations (*n* = 3). All values are expressed on a freeze-dried basis. Different lowercase superscript letters within each processing method and species indicate significant differences among treatment conditions (*p* < 0.05, one-way ANOVA followed by Duncan’s multiple range test). For oven heating and air frying, comparisons were conducted among temperatures at each processing time. Asterisks indicate significant differences from the corresponding untreated control (*** *p* < 0.001; independent-samples *t*-test). Hash symbols indicate significant differences between the two pan-roasting or deep-frying conditions (^###^ *p* < 0.001; independent-samples *t*-test). ND, not detected. CB, common buckwheat; TB, Tartary buckwheat.

## Data Availability

The data presented in this study are available from the corresponding author upon reasonable request.

## Supplementary Materials

The following supporting information can be downloaded at https://www.mdpi.com/article/10.3390/foods15183284/s1. Table S1: Relative contents of rutin and fagopyrin F and their ratio in common and Tartary buckwheat grains after household processing.
