# Supplementary material for: Effects of Household Processing on Rutin and Fagopyrin F in Common and Tartary Buckwheat
Source: Foods. 2026 Sep 17;15(18):3284. doi: 10.3390/foods15183284 (PMC13606827; doi:10.3390/foods15183284)
Supplement: Supplementary file 1 [file foods-15-03284-s001.zip › foods-4541898-supplementary.pdf]

Supplementary Material

Table S1. Relative contents of rutin and fagopyrin F and their ratio in common and Tartary buckwheat grains after household processing.

Processing methods and treatment condition		Relative rutin content (% of control)		Relative fagopyrin F content (% of control)		Rutin-to-fagopyrin F ratio	
		CB	TB	CB	TB	CB	TB
Control	—	100	100	100	100	2.89	172.6
Boiling	3 min	101.2	81.6	48.7	56.2	6.0	250.5
	6 min	99.2	71.5	23.5	52.1	12.2	236.8
	9 min	102.5	50.8	12.5	44.9	23.7	195.4
Steaming	3 min	133.7	93.5	14.2	70.7	27.2	228.3
	6 min	126.8	90.8	15.3	62.4	24.0	251.0
	9 min	129.5	86.0	17.4	40.4	21.4	368.0
Microwave heating	30 s	78.2	100.1	26.0	62.8	8.7	275.2
	60 s	88.6	100.2	23.2	49.6	11.0	348.4
	90 s	92.9	96.9	19.4	44.5	13.8	376.1
	120 s	113.3	95.7	14.7	40.7	22.2	405.8
	150 s	107.8	87.9	9.5	30.1	32.8	503.9
	180 s	127.2	84.3	12.8	26.5	28.8	549.1
Pan roasting	Low flame	103.1	83.3	24.6	52.4	12.1	274.3
	Medium flame	173.3	69.1	ND	17.0	NC	700.0
Oven heating	5 min, 140 °C	97.3	99.9	26.0	71.1	10.8	242.6
	5 min, 160 °C	98.4	97.8	11.4	51.7	24.9	326.3
	5 min, 180 °C	114.2	97.1	9.5	39.0	34.7	430.2
	5 min, 200 °C	140.6	94.5	ND	27.4	NC	596.2
	10 min, 140 °C	96.9	108.7	23.2	66.9	12.0	280.4
	10 min, 160 °C	105.0	98.6	9.5	52.4	31.9	324.5
	10 min, 180 °C	143.4	92.6	8.7	36.2	47.8	441.5
	10 min, 200 °C	164.0	73.4	ND	8.9	NC	1423.7
Deep frying	160 °C	123.7	81.7	10.0	29.5	35.6	478.0
	190 °C	146.8	58.2	9.2	12.1	45.9	826.8
Air frying	5 min, 140 °C	96.8	99.9	19.4	65.9	14.4	261.8

5 min, 160 °C	109.1	101.8	11.4	55.5	27.6	316.5
5 min, 180 °C	120.2	105.9	ND	45.9	NC	398.3
5 min, 200 °C	164.0	91.4	ND	20.7	NC	762.1
10 min, 140 °C	95.5	101.9	16.6	64.5	16.6	272.6
10 min, 160 °C	112.4	104.5	10.9	59.7	29.9	302.2
10 min, 180 °C	123.0	105.7	ND	50.3	NC	362.2
10 min, 200 °C	178.7	92.7	ND	24.6	NC	651.7

Relative rutin and fagopyrin F contents were calculated by dividing the mean content after processing by that of the corresponding untreated control and multiplying by 100. The rutin-to-fagopyrin F ratio was calculated by dividing the mean rutin content by the mean fagopyrin F content on a mass basis. Values are descriptive calculations derived from the group means in Tables 4 and 5. ND, not detected; NC, not calculable because fagopyrin F was not detected; CB, common buckwheat; TB, Tartary buckwheat.
